# A Case of Metastatic Renal Cell Carcinoma Developing Isolated Adrenocorticotropic Hormone (ACTH) Deficiency During Nivolumab Therapy

**DOI:** 10.7759/cureus.99745

**Published:** 2025-12-20

**Authors:** Michihiro Enoki, Kazuaki Yamanaka, Atsunari Kawashima, Shinichiro Fukuhara, Norio Nonomura

**Affiliations:** 1 Department of Urology, Osaka University Graduate School of Medicine, Suita, JPN

**Keywords:** immune-related adverse event (irae), isolated acth deficiency, metastatic renal cell carcinoma, pd -1 inhibitor, secondary adrenal insufficiency

## Abstract

We report a case of isolated adrenocorticotropic hormone (ACTH) deficiency that developed after nivolumab therapy for metastatic renal cell carcinoma involving the pancreas and thyroid. The patient presented with nonspecific symptoms that improved promptly after discontinuation of nivolumab and initiation of hydrocortisone replacement therapy. Despite permanently requiring glucocorticoid supplementation, the patient has maintained a durable partial response for more than four years after cessation of nivolumab. This case highlights the importance of early recognition of immune-related endocrinopathies and demonstrates that long-term tumor control may be achieved even after discontinuation of immune checkpoint inhibitors.

## Introduction

Programmed cell death-1 (PD-1) inhibitors are widely used as standard therapeutic agents for metastatic renal cell carcinoma; however, they are associated with a broad spectrum of immune-related adverse events (irAE) that are often difficult to predict. Among these, isolated adrenocorticotropic hormone (ACTH) deficiency induced by anti-PD-1 antibodies is rare, with a reported incidence of less than 1% [1]. Although uncommon, ACTH deficiency leading to secondary adrenal insufficiency can be life-threatening and therefore requires prompt recognition and appropriate management. Here, we report a case of isolated ACTH deficiency with secondary adrenal insufficiency that developed during nivolumab therapy for metastatic renal cell carcinoma.

## Case presentation

A 67-year-old Japanese woman presented with metastatic clear cell renal cell carcinoma involving the thyroid and pancreas. She underwent laparoscopic nephrectomy, and first-line treatment with sunitinib was initiated; however, therapy was discontinued due to palmar-plantar erythrodysesthesia syndrome and hepatotoxicity. Nivolumab was subsequently introduced as a second-line treatment.

Eight months after nivolumab initiation, she presented with a two-week history of fatigue and anorexia. Her vital signs revealed hypotension (systolic blood pressure, 95 mmHg) and tachycardia (heart rate, 100 bpm). Laboratory tests showed eosinophilia (23.5%), while no significant electrolyte abnormalities or hypoglycemia were observed. Endocrinological evaluation revealed low adrenocorticotropic hormone (2 pg/mL) and low serum cortisol levels (1.1 µg/dL). Other pituitary hormone levels were within normal ranges, excluding thyroid dysfunction, hypophysitis with multiple pituitary hormone deficiencies, and central hypogonadism (Table 1). Further dynamic endocrine testing, including adrenocorticotropic hormone stimulation, corticotropin-releasing hormone stimulation, and insulin tolerance tests, was performed to differentiate adrenal insufficiency and hypothalamic-pituitary-adrenal axis disorders, leading to the diagnosis of isolated ACTH deficiency and secondary adrenal insufficiency. Pituitary magnetic resonance imaging showed no structural abnormalities.

**Table 1:** Circulating Endocrine Profile TSH: Thyroid-stimulating hormone, FT4: Free thyroxine, GH: Growth hormone, PRL: Prolactin, LH: Luteinizing hormone, FSH: Follicle-stimulating hormone

Parameters	Patient Values	Reference Range
ACTH (pg/mL)	2.0	7.0–63.0
cortisol (µg/dL)	1.1	4.0–18.3
TSH (µIU/mL)	4.43	0.45–3.72
FT4 (ng/dL)	1.4	0.8–1.7
GH (ng/mL)	0.95	0.13–9.88
PRL (ng/mL)	64.7	4.1–27.9
LH (mIU/mL)	16.1	1.1–8.1
FSH (mIU/mL)	75.1	4.0–14.2

Hydrocortisone (50 mg/day) was initiated, resulting in rapid improvement of symptoms. The dosage was gradually tapered to 15 mg/day without recurrence.

Nivolumab was reintroduced one month after the initiation of hormone replacement therapy. Ten months later, following an episode of upper respiratory infection, the patient again developed fatigue and anorexia. Relative adrenal insufficiency due to increased physiological cortisol demand (sick-day condition) was suspected. Hydrocortisone was temporarily increased to 45 mg/day, and nivolumab was discontinued. After recovery, hydrocortisone was tapered back to 15 mg/day, with no recurrence of symptoms.

**Figure 1:**
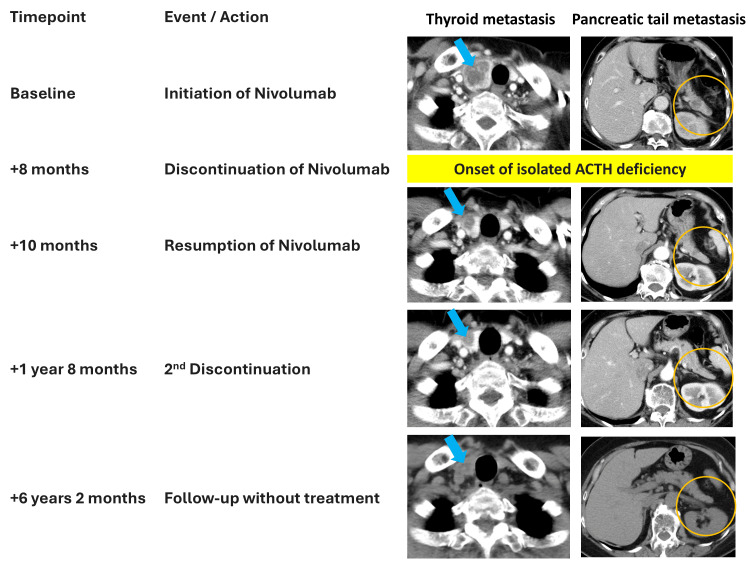
Clinical course and tumor response to nivolumab therapy All images represent axial sections of contrast-enhanced CT from the neck to the abdomen, except for the final panel, which shows a non-contrast CT covering the same regions. Serial imaging demonstrates metastatic lesions in the thyroid and pancreas at baseline, reduction in tumor size following nivolumab initiation, and sustained partial response after discontinuation of therapy. The timeline includes the onset of isolated ACTH deficiency, hydrocortisone replacement, nivolumab re-initiation, and eventual cessation.

Metastatic lesions in the pancreas and thyroid decreased in size after nivolumab administration, and a partial response has been maintained for more than 4.5 years without further systemic therapy (Figure 1).

## Discussion

Immune checkpoint inhibitors (ICIs), including anti-PD-1, anti-programmed death-ligand 1 (PD-L1), and anti-cytotoxic T lymphocyte-associated antigen-4 (CTLA-4) antibodies, are known to cause immune-related hypophysitis and associated hormonal deficiencies. According to a recent meta-analysis, the incidence of hypophysitis is <0.1% with anti-PD-L1 antibodies, 0.4% with anti-PD-1 antibodies, 3.2% with anti-CTLA-4 antibodies, and 6.4% with combination therapy [2]. Conversely, a prospective study from Japan reported higher incidences: 6.0% with anti-PD-1 antibodies and 24.0% with anti-CTLA-4 antibodies [3].

ICI-induced hypopituitarism manifests in two major patterns: (1) hypophysitis, characterized by pituitary enlargement and multiple anterior pituitary hormone deficiencies, including ACTH, and (2) isolated ACTH deficiency without pituitary enlargement. Anti-PD-1/PD-L1 antibodies more commonly cause isolated ACTH deficiency, whereas anti-CTLA-4 antibodies can cause either phenotype. Moreover, hypophysitis associated with anti-CTLA-4 antibodies tends to occur earlier. These differences are attributed to CTLA-4 expression in pituitary tissue and the ability of ipilimumab (IgG1) to activate complement pathways and induce T-cell infiltration [4-6]. In contrast, nivolumab and pembrolizumab (IgG4) have weaker complement-binding capacity, and PD-1/PD-L1 expression in the pituitary is not clearly established (Table 2) [7].

**Table 2:** Characteristics of Immune Checkpoint Inhibitor-Induced Hypopituitarism This table was created by the authors.

Category	Anti-PD-1/Anti-PD-L1 Antibodies	Anti-CTLA-4 Antibodies
Incidence	0.1–13.3%	1.5–24.0%
Predominant Pattern	Mostly isolated ACTH deficiency	Hypophysitis with multiple anterior pituitary hormone deficiencies (ACTH, TSH, gonadotropins)
Median Time to Onset (Range)	25.8 weeks (18.4–44.0 weeks)	9.3 weeks (7.2–11.1 weeks)
Mechanistic Features	Pituitary PD-L1 expression unclear; mostly IgG4 or Fc-modified IgG1; weak complement activation	CTLA-4 expression in the pituitary; IgG1 antibody induces complement activation and T-cell infiltration
Representative References	[1,3,15]	[2,3,5]

Diagnosis is often delayed because symptoms such as fatigue and anorexia are nonspecific. Although thyroid function tests are routinely performed during ICI therapy, screening for pituitary dysfunction is not consistently practiced [8]. Regular endocrine evaluation is therefore critical for timely diagnosis [9]. Most patients require lifelong hydrocortisone replacement, as was the case in our patient [10]. Importantly, ICI therapy may be continued if symptoms are controlled with adequate glucocorticoid replacement [11].

The association between immune-related adverse events and improved oncologic outcomes has been reported in several malignancies, including renal cell carcinoma, lung cancer, and melanoma [12,13]. In renal cell carcinoma, all five reported patients who developed isolated ACTH deficiency during nivolumab therapy demonstrated tumor shrinkage or disease stabilization after treatment cessation [14]. In our case, despite discontinuation of nivolumab due to recurrent adrenal insufficiency, the patient has maintained a partial response for more than four years, supporting a potential association between endocrine irAEs and a favorable prognosis.

## Conclusions

We report a case of isolated ACTH deficiency and secondary adrenal insufficiency that developed following nivolumab therapy for metastatic renal cell carcinoma. During treatment with anti-PD-1 or anti-PD-L1 antibodies, careful assessment of symptoms and regular endocrine monitoring are essential for the early detection of hypopituitarism and secondary adrenal insufficiency.

## References

[1] Arima H, Iwama S, Inaba H (2019). Management of immune-related adverse events in endocrine organs induced by immune checkpoint inhibitors: clinical guidelines of the Japan Endocrine Society. Endocr J.

[2] Barroso-Sousa R, Barry WT, Garrido-Castro AC, Hodi FS, Min L, Krop IE, Tolaney SM (2018). Incidence of endocrine dysfunction following the use of different immune checkpoint inhibitor regimens: a systematic review and meta-analysis. JAMA Oncol.

[3] Kobayashi T, Iwama S, Yasuda Y (2020). Pituitary dysfunction induced by immune checkpoint inhibitors is associated with better overall survival in both malignant melanoma and non-small cell lung carcinoma: a prospective study. J Immunother Cancer.

[4] Iwama S, De Remigis A, Callahan MK, Slovin SF, Wolchok JD, Caturegli P (2014). Pituitary expression of CTLA-4 mediates hypophysitis secondary to administration of CTLA-4 blocking antibody. Sci Transl Med.

[5] Tsoli M, Kaltsas G, Angelousi A, Alexandraki K, Randeva H, Kassi E (2020). Managing ipilimumab-induced hypophysitis: challenges and current therapeutic strategies. Cancer Manag Res.

[6] Caturegli P, Di Dalmazi G, Lombardi M (2016). Hypophysitis secondary to cytotoxic T-lymphocyte-associated protein 4 blockade: insights into pathogenesis from an autopsy series. Am J Pathol.

[7] Di Dalmazi G, Ippolito S, Lupi I, Caturegli P (2019). Hypophysitis induced by immune checkpoint inhibitors: a 10-year assessment. Expert Rev Endocrinol Metab.

[8] Darapu H, Konindala N, Paluri R (2025). Checkpoint on adrenal insufficiency: optimizing screening in immune checkpoint inhibitor therapy. Cureus.

[9] Levy M, Abeillon J, Dalle S (2020). Anti-PD1 and anti-PDL1-induced hypophysitis: a cohort study of 17 patients with longitudinal follow-up. J Clin Med.

[10] Albarel F, Gaudy C, Castinetti F (2015). Long-term follow-up of ipilimumab-induced hypophysitis, a common adverse event of the anti-CTLA-4 antibody in melanoma. Eur J Endocrinol.

[11] Verzoni E, Cartenì G, Cortesi E (2019). Real-world efficacy and safety of nivolumab in previously-treated metastatic renal cell carcinoma, and association between immune-related adverse events and survival: the Italian expanded access program. J Immunother Cancer.

[12] Yoshimura A, Kato T, Nakai Y (2024). Clinical outcomes of first-line combination therapy with immune checkpoint inhibitor for metastatic non-clear cell renal cell carcinoma: a multi-institutional retrospective study in Japan. Int J Clin Oncol.

[13] Paderi A, Giorgione R, Giommoni E (2021). Association between immune related adverse events and outcome in patients with metastatic renal cell carcinoma treated with immune checkpoint inhibitors. Cancers (Basel).

[14] Suzuki K, Terakawa T, Furukawa J, Harada K, Hinata N, Nakano Y, Fujisawa M (2020). Nivolumab-induced adrenal insufficiency in patients with renal cell carcinoma. J Immunother.

[15] Faje A, Reynolds K, Zubiri L (2019). Hypophysitis secondary to nivolumab and pembrolizumab is a clinical entity distinct from ipilimumab-associated hypophysitis. Eur J Endocrinol.

